# *Acanthopanax senticosus* improves cognitive impairment in Alzheimer’s disease by promoting the phosphorylation of the MAPK signaling pathway

**DOI:** 10.3389/fimmu.2024.1383464

**Published:** 2024-03-13

**Authors:** Zhichun Zhang, Yonghui Wu, Dan Shi, Chanyu Jiang, Hengyan Cao, Fengyi Jiang, Xiaomin Bao, Yan Shen, Xiao Shi

**Affiliations:** ^1^ Department of Gerontology, Yueyang Hospital of Integrated Traditional Chinese and Western Medicine, Shanghai, China; ^2^ Graduate School of Shanghai University of Traditional Chinese Medicine, Shanghai, China

**Keywords:** *Acanthopanax senticosus*, Alzheimer’s disease, network pharmacology, MAPK signaling pathway, neuroinflammation

## Abstract

**Background:**

*Acanthopanax senticosus* (AS) can improve sleep, enhance memory, and reduce fatigue and is considered as an effective drug for Alzheimer’s disease (AD). The therapeutic effect and mechanism need to be further investigated.

**Methods:**

To confirm the AS play efficacy in alleviating memory impairment in mice, 5×FAD transgenic mice were subjected to an open-field experiment and a novelty recognition experiment. Network pharmacology technique was used to analyze the information of key compounds and potential key targets of AS for the treatment of AD, molecular docking technique was applied to predict the binding ability of targets and compounds, and Gene Ontology (GO) and Kyoto Encyclopedia of Genes and Genomes (KEGG) analyses were also performed on the targets to derive the possible metabolic processes and pathway mechanisms of AS in treating AD. Quantitative real-time PCR (qRT-PCR) and western blot technique were carried out to validate the candidate genes and pathways.

**Results:**

In the open-field experiment, compared with the wild-type (WT) group, the number of times the mice in the AD group crossed the central zone was significantly reduced (*P*< 0.01). Compared with the AD group, the number of times the mice in the AS group crossed the central zone was significantly increased (*P*< 0.001). In the new object recognition experiment, compared with the WT group, the percentage of times the AD group explored new objects was significantly reduced (*P*< 0.05). Compared with the AD group, the AS group had an increase in the percentage of time spent exploring new things and the number of times it was explored (*P*< 0.05). At the same time, the donepezil group had a significantly higher percentage of times exploring new things (*P*< 0.01). By using network pharmacology technology, 395 common targets of AS and AD were retrieved. The Cytoscape software was used to construct the protein–protein interaction (PPI) network of common targets. Using the algorithm, nine key targets were retrieved: APP, NTRK1, ESR1, CFTR, CSNK2A1, EGFR, ESR2, GSK3B, and PAK1. The results of molecular docking indicate that 11 pairs of compounds and their corresponding targets have a significant binding ability, as the molecular binding energies were less than -7.0. In comparison to the AD group, the mRNA expression of the key target genes was significantly decreased in the AS treatment group (*P*< 0.001). The KEGG analysis showed that the MAPK signaling pathway was significantly enriched, and Western blot confirmed that the TRAF6 protein decreased significantly (*P*< 0.0001). Meanwhile, the levels of MAP3K7 and P38 phosphorylation increased, and there was also an increase in the expression of HSP27 proteins.

**Conclusion:**

Our study indicates that the multi-component and multi-target properties of AS play an important role in the alleviation of anxiety and memory impairment caused by AD, and the mechanism is involved in the phosphorylation and activation of the MAPK signaling pathway. The results of this study could provide a novel perspective for the clinical treatment of AD.

## 1 Introduction

Alzheimer’s disease (AD) is the most common neurodegenerative disease which is a common cause of dementia, and the incidence of AD gradually increases with age (1). The clinical manifestations include memory loss, disorientation, and language impairment. The patient’s ability to handle social affairs and self-care will decline as the condition worsens (2). Current hypothesis for the pathogenesis of AD include Aβ plaque-related neurodegeneration, neurofibrillary tangles, synaptic dysfunction and neurotransmitter imbalance, and neuroinflammation (3). FDA-approved drugs including donepezil, rivastigmine, and galantamine are mainly used to treat β-amyloid deposition and tau fiber tangles, but the effect is not significant (3, 4). More and more studies showed that neuroinflammation played a key role in AD neurodegeneration, and how to control the inflammatory response caused by Aβ protein provides a new research direction for the treatment of AD (5–7).

Related research showed (8–10) that traditional Chinese medicine with multiple components, targets, and pathways can treat AD by improving neurocholine function, reducing inflammatory response, and resisting oxidative stress. *Acanthopanax senticosus* (AS) is a Wujiaceae plant that can improve sleep, enhance memory, and reduce fatigue (11). Relevant experimental studies have shown that AS extract can significantly enhance mice’s object recognition memory (12); EEAK (ethanol extract of AS) can improve cognitive dysfunction caused by cholinergic blockade and improve the performance of mice in Y-maze and novel object recognition experiments (13). ML Jinc et al. found that (14) AS can induce the expression of HO-1 through the p38-CREB and Nrf2 pathways, thereby reducing the expression of pro-inflammatory mediators such as iNOS, COX-2, and NO in LPS-stimulated BV2 cells, and has a neuroprotective effect. Therefore, AS is considered to have a certain therapeutic effect on AD, but its possible mechanism is still unclear.

This study intends to verify the therapeutic effect of the traditional Chinese medicine AS on AD through system network pharmacology, molecular docking technology, and animal experiments, explore the relevant molecular mechanisms, and provide more scientific basis for the clinical treatment of AD.

## 2 Materials and methods

### 2.1 Preparation and feeding of experimental animal models

Specific-pathogen-free (SPF)-grade 5×FAD transgenic mice were obtained from the Model Animal Research Institute of Nanjing University (Animal Qualification Certificate No. 201400975) and kept under SPF conditions. The animal experiments were approved by the Animal Care and Use Committee of Shanghai University of Traditional Chinese Medicine (ethics number: PZSHUTCM210702001).

5×FAD transgenic mice were cross-bred with C57BL/6J mice. The mice were raised in separate cages according to their gender after 20 days of life. Their tails were docked at 30 days for genetic identification of mouse breeds, and 5×FAD mice were selected as the *Acanthopanax senticosus* treatment group (AS group), the donepezil hydrochloride treatment group (donepezil group), and the model group (AD group), while the C57BL/6J mice in the same litter were the blank group (WT group), with 12 mice in each group. After 2 months of regular feeding of the mice, the AS group was changed to a diet supplemented with 1.69 mg/kg of *Acanthopanax senticosus* (Heilongjiang Ussuri River Harbin Branch, batch no. 20210501), and the donepezil group was changed to a diet containing 3.8 g/kg of the drug, donepezil hydrochloride tablets (Sibohai, Phyllanthus Bio-Technology Co. Ltd, batch no. 21030004). All the drug-containing diets were manufactured by Jiangsu Synergy Pharmaceutical and Biological Engineering Co. Ltd., and animal tissues were acquired after 3 months of continuous feeding.

### 2.2 Animal experiment

#### 2.2.1 Open-field experiment

The test box for the open-field experiment consisted of four square opaque boxes with a length, width, and height dimension of 50 × 50 × 40 cm, and a square area of 20×20 cm in the center of the test box was designated as the analysis area. Each mouse was allowed to move freely in the test box for 5 min, and the total distance traveled, the average speed of movement, and the number of times the mice crossed the central area of the experimental site were recorded by using Ethovision XT 11.5 image acquisition and analysis software for the different groups. The experimental environment was kept quiet, and the test chamber was wiped with 75% alcohol after each round of experiments.

#### 2.2.2 Novelty recognition experiment

The test chambers for the novelty recognition experiment consisted of four square opaque whiteboards measuring 50 × 50 × 40 cm in length, width, and height, with two identical X-objects and Y-objects placed in the center of each chamber and secured with a transparent tape. The detection behavior of each mouse was recorded for 5 min using Ethovision XT 11.5 image acquisition and analysis software. Approximately 1 h after the abovementioned behavioral experiments, object Y was replaced by object Z, which was different in size, shape, and color from object Y, and then the mice were placed in the experimental field in the order of the sequence and moved randomly around the experimental field for 5 min. The residence time and number of times the mice explored each object were recorded. The location preference index of the first trial was calculated as RI = Tx/(Ty + Tx) × 100%, which was used to observe whether the experimental mice had a preference for toys X and Y, and whether there was a difference in curiosity between the toys, to determine whether the curious nature of the mice’s exploration was normal. The formula for the experimental position preference index for the next 1 h is RI = Tz/(Tz + Tx) × 100%, which was used to determine the curiosity index of the experimental mice toward the novel object Z.

### 2.3 Network pharmacology analysis

#### 2.3.1 Component collection and target prediction

The TCMIP (http://www.tcmip.cn/TCMIP/index.php/Home/Login/login.html) and HERB (http://herb.ac.cn/) databases were searched using the keyword “ciwujia” (*Acanthopanax senticosus*, AS). The screening conditions were oral bioavailability (OB) ≥0.3, drug likeness (DL) ≥0.18, Lipinsk’s five principles, and high gastrointestinal absorption, and we supplemented the literature with active ingredients that have clear utility in AS (15). The Swiss Target Prediction database (http://www.swisstargetprediction.ch/) was used to predict the targets of the relevant compounds. Using “Alzheimer’s disease” as a keyword, the OMIM database (https://www.omim.org/), TTD database (http://db.idrblab.net/ttd/), and GeneCards database (https://www.genecards.org/) (score ≥5) were searched, screened, and intersected to obtain AD-related disease targets.

#### 2.3.2 Component–target network construction of AS treatment for AD

The targets of AS and AD were imported into the Venny 2.1 online software mapping tool platform (https://bioinfogp.cnb.csic.es/tools/venny/) to draw a Venn diagram, and the common targets obtained were the potential targets of AS for AD. These potential targets and their corresponding compound data were processed with Cytoscape 3.7.2 software to construct a compound–target network (CTN) of AS for AD.

#### 2.3.3 Construct protein–protein interaction network

Taking the target in the abovementioned CTN as the objective, IntAct, BioGrid, and STRING databases are used to construct the protein–protein (PPI) network. The MCODE algorithm was used to filter the key clusters in the PPI network, construct the PPI network, and calculate the parameters of degree, degree centrality (DC), closeness centrality (CC), and betweenness centrality (BC) of the network and then select the core clusters according to the mean value.

#### 2.3.4 GO enrichment analysis and KEGG pathway analysis

The DAVID database (https://david.ncifcrf.gov/) was used to perform Gene Ontology (GO) functional annotation of core targets, including Biological Process (BP), Cellular Component (CC), and Molecular Function (MF), and Kyoto Encyclopedia of Genes and Genomes (KEGG) enrichment analysis was performed to predict the possible pathway mechanisms of AS for AD.

### 2.4 Molecular docking

The core targets obtained from the PPI network were mapped to the CTN network to find the key compounds corresponding to the core targets. The 3D protein structure of the core target was retrieved from the PDB database (https://www.rcsb.org/) as a receptor, and the receptor protein was subjected to pre-docking preparation operations such as dehydrogenation and hydrogenation by UCSF Chimera 1.16 software. Using the abovementioned key compounds as ligands, molecular docking was performed using Auto Dock Vina 1.1.2 software, and the pairs with binding energies ≤-7.5 were visualized by Pymol 2.4.0 software.

### 2.5 qRT-PCR detection of key protein mRNA content

mRNA was extracted from the cerebral cortex of each group of mice using the TRIzol method. cDNA reverse transcription was performed at 25°C for 5 min, 55°C for 10 min, and 85°C for 5 s. The PCR primers were purchased from Sangong Bioengineering (Shanghai) Co., Ltd., and the sequence of the primers (5′ to 3′) is shown in **Table 1**. The reaction program was set at 95°C for 30 s, 95°C for 15 s, and 60°C for 30 s in the quantitative PCR instrument (C1000 Touc, BIO-RAD). The results were expressed as 2^-ΔΔCT^ values and analyzed for statistical differences.

**Table 1:** qRT-PCR primer sequences.

Gene	Forward (o′– 3′)	Reverse (e′– 3′)
GAPDH	AGGTCGGTGTGAACGGATTTG	TGTAGACCATGTAGTTGAGGTCA
APP	ACCCCAGATCGCCATGTTC	CCCACTTTCCATTCTGCACATTC
NTRK1	CAGTCTGATGACTTCGTTGATGC	CTCTTCACGATGGTTAGGCTTC
ESR1	CCCGCCTTCTACAGGTCTAAT	CTTTCTCGTTACTGCTGGACAG
CFTR	CTGGACCACACCAATTTTGAGG	GCGTGGATAAGCTGGGGAT
CSNK2A1	ATGTGGTGGAATGGGGGAATC	GCAAGTGTGATGATGTTGGGC
EGFR	GCATCATGGGAGAGAACAACA	TCAGGAACCATTACTCCATAGGT
ESR2	CTGTGCCTCTTCTCACAAGGA	TGCTCCAAGGGTAGGATGGAC
GSK3B	AAGCGATTTAAGAACCGAGAGC	AGAAATACCGCAGTCGGACTAT
PAK1	GAAACACCAGCACTATGATTGGA	ATTCCCGTAAACTCCCCTGTG

GAPDH, glyceraldehyde-3-phosphate dehydrogenase; APP, amyloid precursor protein; NTRK1, neurotrophic receptor tyrosine kinase 1; ESR1, estrogen receptor 1; CFTR, CF transmembrane conductance regulator; CSNK2A1, casein kinase 2 alpha 1; EGFR, epidermal growth factor receptor; ESR2, estrogen receptor 2; GSK3B, glycogen synthase kinase 3 beta; PAK1, P21 (RAC1) activated kinase 1.

### 2.6 Western blot to validate the pathway of action

An appropriate amount of mouse hippocampal tissue stored at -80°C in the refrigerator was taken out, RIPA lysis buffer was added, and the tissue was disrupted with an ultrasonic crusher and centrifuged at 12,000 rpm for 15 min. The supernatant was aspirated and 5× Protein Loading Buffer (Beyotime, P0015) was added. The protein samples were heated in a water bath at 100°C for 10 min. Electrophoresis was performed at 120 V for 80 min, and the membranes were transferred to a new membrane. After blocking with 5% BSA solution for 2 h, the primary antibody was incubated. The antibodies used for each sample were TRAF6 (#8028, CST, 1:1,000), anti-MAP3K7 (K008561P, Solarbio, 1:1,000), anti-p-MAP3K7 (K006194P, Solarbio, 1:1,000), P38 (#8690, CST, 1: 1,000), p-P38 (#4511, CST, 1:1,000), and HSP27 (#95357, CST, 1:1,000), overnight at 4°C in the refrigerator. After three washes in TBST solution, secondary antibodies were used: anti-rabbit IgG, HRP-conjugated antibody (#7074, CST, 1:2,000) was incubated for 1 h. The protein band signals were captured using the FL1000 Intelligent Imaging Scanning System (Thermo Fisher Scientific, USA). The protein band intensities were quantified using Image J (v1.45f) software.

### 2.7 Statistical method

The experimental data obtained from all behavioral experiments were expressed as mean ± SD $\left(\bar{x}\pm s\right)$ , and SPSS 26.0 software was used for statistical analysis. One-way ANOVA was used for two-way comparisons between multiple groups and when *P<*0.05 is a statistically significant difference criterion.

## 3 Results

### 3.1 Behavioral experiment results

**Figure 1:**
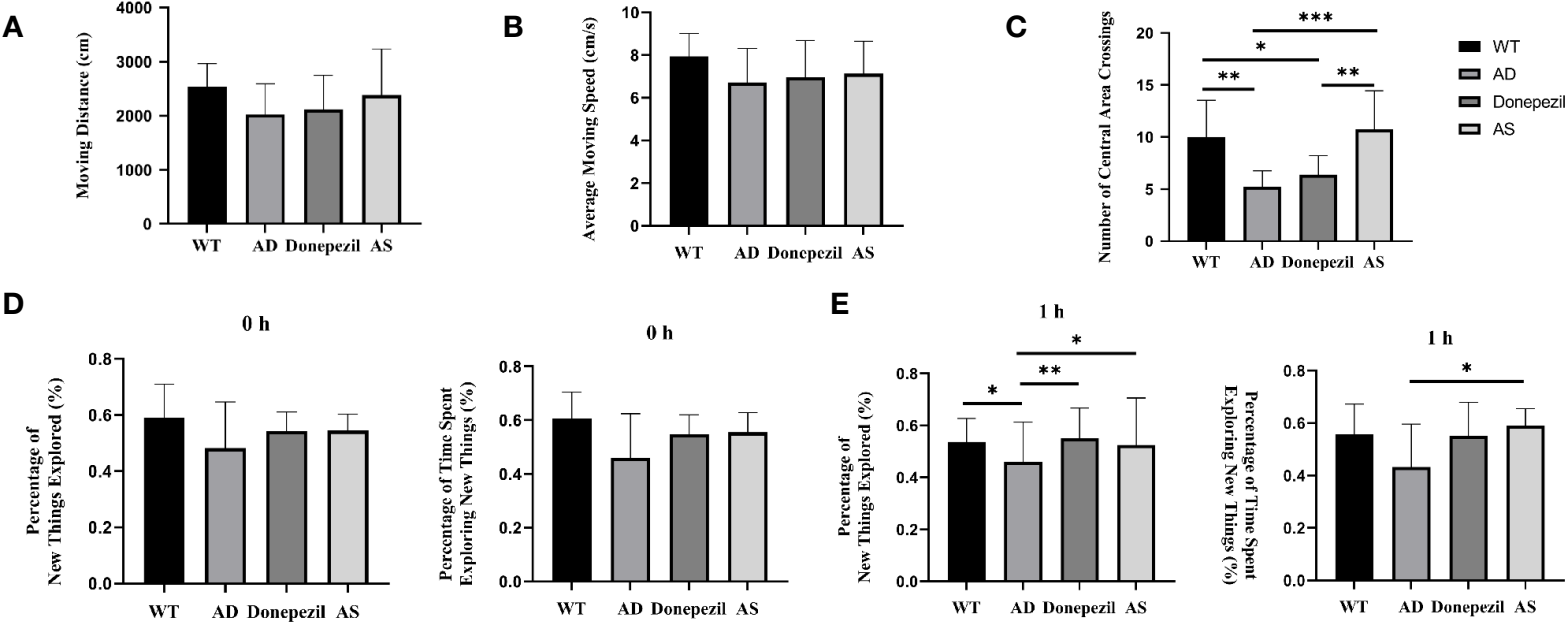
Behavioral experimental results on the open-field test and the new-object recognition experiment. **(A)** Moving distance in each group of the open-field test. **(B)** Average moving speed in each group of the open-field test. **(C)** Times of crossing the central area in each group of the open-field test. **(D)** 0 h percentage of new things explored and 0 h percentage of time exploring new things. **(E)** 1 h percentage of new things explored and 1 h percentage of time exploring new things (*n* = 8). ^*^ *P* < 0.05, ^**^ *P* < 0.01, ^***^ *P* < 0.001.

The effects of AS on anxiety, nervousness, and other emotions of mice were analyzed by recording the distance traveled, average speed of movement, and number of times crossing the central zone of different groups of freely moving mice in the open-field experiment, and the results show that there was no significant difference between the distance traveled and the average speed of movement of mice in the WT, AD, donepezil, and AS groups in the behavioral experiments (*P*< 0.05) (**Figures 1A, B**). The number of times the mice in the AD group crossed the central zone was significantly lower than that of the WT group (5.38 ± 1.19 vs. 10.12 ± 3.31; *P<* 0.01), while the number of times the mice in the AS group traversed the central zone was significantly higher than that of the AD group (11.38 ± 3.11 vs. 5.38 ± 1.19; *P*< 0.001), whereas there was no significant difference in the donepezil group (6.25 ± 1.75 vs. 5.38 ± 1.19) (**Figure 1C**).

Mice are naturally curious and exploratory of new things, and the effect of spikenard on learning and memory impairment in demented mice could be determined by conducting new object recognition experiments. The experimental results showed that in the training phase (0 h) (**Figure 1D**), there was no significant difference in the percentage of time spent exploring unfamiliar toys (0.60 ± 0.05 vs. 0.53 ± 0.11 vs. 0.55 ± 0.06 vs. 0.57 ± 0.04; *P* > 0.05) and the percentage of number of explorations (0.57 ± 0.07 vs. 0.54 ± 0.11 vs. 0.56 ± 0.07 vs. 0.60 ± 0.04; *P* > 0.05) among the mice in the WT, AD, donepezil, and AS groups, indicating that there was no difference in the inherent curiosity for novelty among the mice in each group.

During the test phase (1 h) (**Figure 1E**), compared with the WT group, the AD group showed a decrease in the percentage of number of times of curious exploration of object Z (0.45 ± 0.07 vs. 0.55 ± 0.07; *P*< 0.05) and a decrease in the percentage of time spent exploring (0.46 ± 0.09 vs. 0.56 ± 0.05), but there was no significant difference; compared with AD group, the percentage of time spent exploring and the percentage of number of times of curious exploration of object Z are increased in the AS group (0. 56 ± 0.08 vs. 0.45 ± 0.07; 0.60 ± 0.07 vs. 0.46 ± 0.09; *P*< 0.05); compared with the AD group, the donepezil group had a significantly higher percentage of times exploring object Z (0.58 ± 0.05 vs. 0.45 ± 0.07; *P*< 0.01) and an increased percentage of time exploring object Z, but there was no statistically significant difference and it was lower than the AS group.

### 3.2 Active ingredients–target network of AS in treating AD

**Figure 2:**
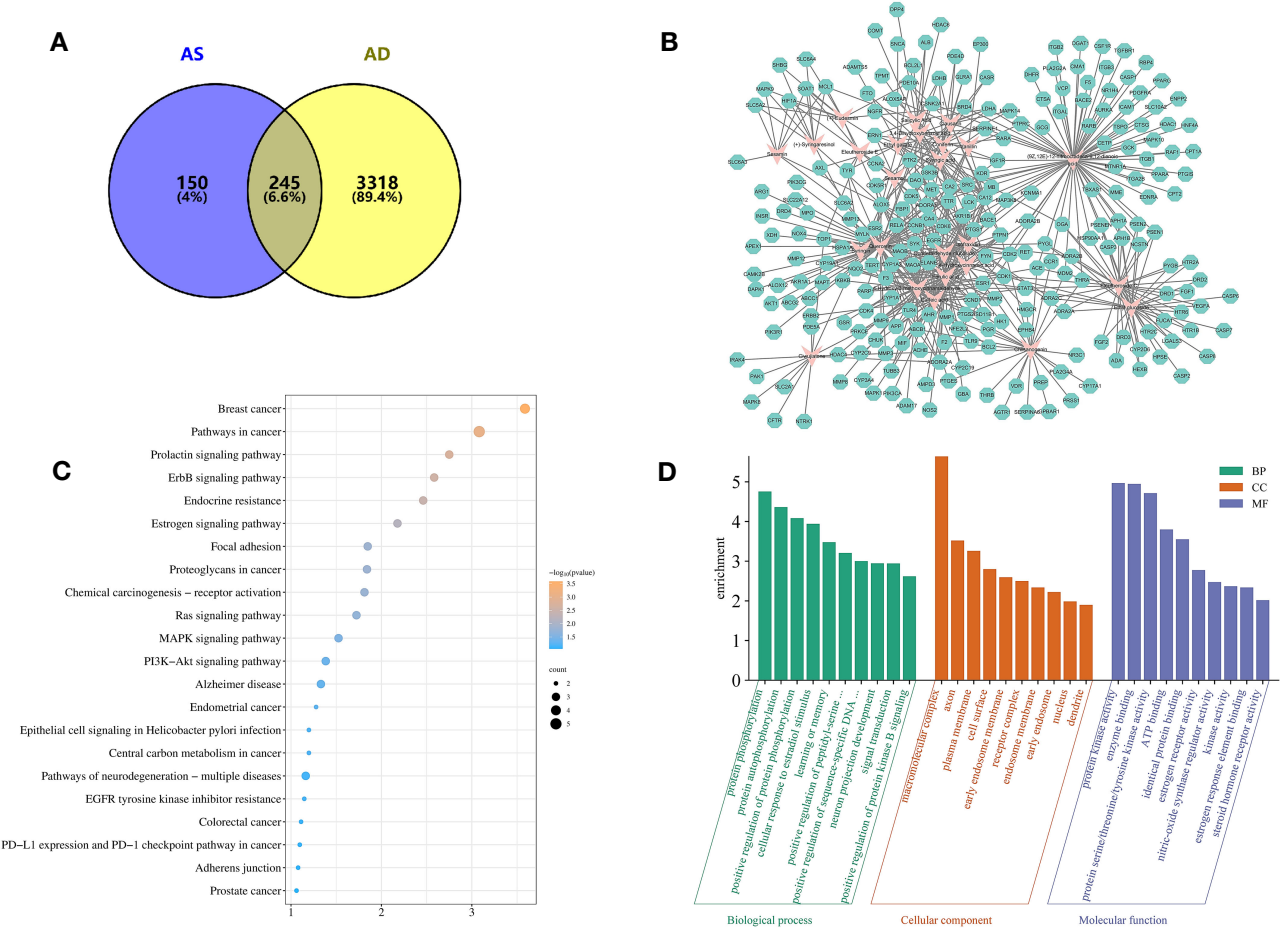
Target information and network topology analysis results of *Acanthopanax senticosus* (AS) and Alzheimer’s disease. **(A)** Venn diagram. **(B)** “Compound–target” network (CTN) of common targets and compounds in AS. Pink rhombus, compounds; green polygons, targets. **(C)** Kyoto Encyclopedia of Genes and Genomes pathways enrichment analysis. The bluer the color, the larger the *P* value. The size of each bubble reflects the number of genes enriched in the terms. **(D)** Gene Ontology enrichment analysis.

By searching the TCMIP database, the HERB database, and the Chinese Pharmacopoeia (2020 edition), 25 active ingredients (**Table 2**) and 395 targets for AS were obtained. GeneCards database (score ≥5), OMIM database, and TTD database were used to retrieve 3563 AD-related targets. The intersection of the two databases yielded 245 targets common to AS and AD (**Figure 2A**), which belonged to a total of 25 chemical components in AS, and a compound–target network (CTN) was constructed using Cytoscape-v3.7.2 software (**Figure 2B**).

**Table 2:** Potentially active compounds in *Acanthopanax senticosus*.

PubChem CID	Compound
72	3,4-Dihydroxybenzoic acid
338	Salicylic acid
1183	Vanillin
10742	Syringic acid
13250	Ethyl gallate
73117	(+)-Eudesmin
15699109	Coniferaldehyde glucoside
21636080	Chiisanogenin
428040	Ethyl glucoside
443023	(+)-Syringaresinol
445858	Ferulic acid
5280343	Quercetin
5280372	Coniferin
5280536	4-Hydroxy-3-methoxycinnamaldehyde
5282316	(9Z,12E)-12-Nitrooctadeca-9,12-dienoic acid
5315944	Ciwujiatone
5315945	Clausarin
5316860	Syringin
5318565	Isofraxidin
637542	4-Hydroxycinnamic acid
68289	Sesamo
689043	Caffeic acid
71312557	Eleutheroside E
72307	Sesamin
9859136	Eleutheroside C

### 3.3 Analysis of core targets and biological functions of AS in treating AD

Protein interaction pairs (PPI pairs) of the abovementioned 245 targets screened in IntAct (https://www.ebi.ac.uk/intact/), BioGrid (https://thebiogrid.org/), and STRING databases were transferred to Cytoscape software to construct the protein–protein interaction (PPI) network for AS of AD. The topological parameters of each node in the PPI network were calculated using the MCODE algorithm, and a total of 19 core protein interaction cluster networks were screened, of which 85 targets related to AS for AD were identified in these 19 core protein interaction clusters. The core nodes were screened by the mean values of degree, degree centrality (DC), closeness centrality (CC), betweenness centrality (BC) and other parameters, and finally nine key targets were obtained, including APP, NTRK1, ESR1, CFTR, CSNK2A1, EGFR, ESR2, GSK3B, and PAK1, which suggest that these targets play an important role in AS for AD.

The results of the GO enrichment analysis of these nine key targets showed that (**Figure 2D**) the biological process (BP) of AS treatment of AD was highlighted in the processes of protein phosphorylation, protein autophosphorylation, positive regulation of protein phosphorylation, cellular response to estradiol stimulation, and learning or memory; cellular composition (CC) was mainly in the processes of macromolecular complexes, axons, plasma membrane, cell surface, early endosomal membranes and receptor complexes, etc.; and molecular function (MF) focuses on the role of enzyme binding, protein serine/threonine/tyrosine kinase activity, ATP binding, binding of the same proteins, estrogen receptor activity, and nitric oxide synthase regulatory activity.

The KEGG pathway analysis identified 22 relevant biological pathways that may be significantly affected by AS in the treatment of AD (**Figure 2C**). AS was found to play a role in some cancer pathways, such as breast and endometrial cancer as well as in Alzheimer’s disease pathways and pathways associated with neurodegenerative diseases. Others play important roles in the ErbB signaling pathway, the Ras signaling pathway, the MAPK signaling pathway, the PI3K-Akt signaling pathway, and so on.

### 3.4 Molecular docking

**Figure 3:**
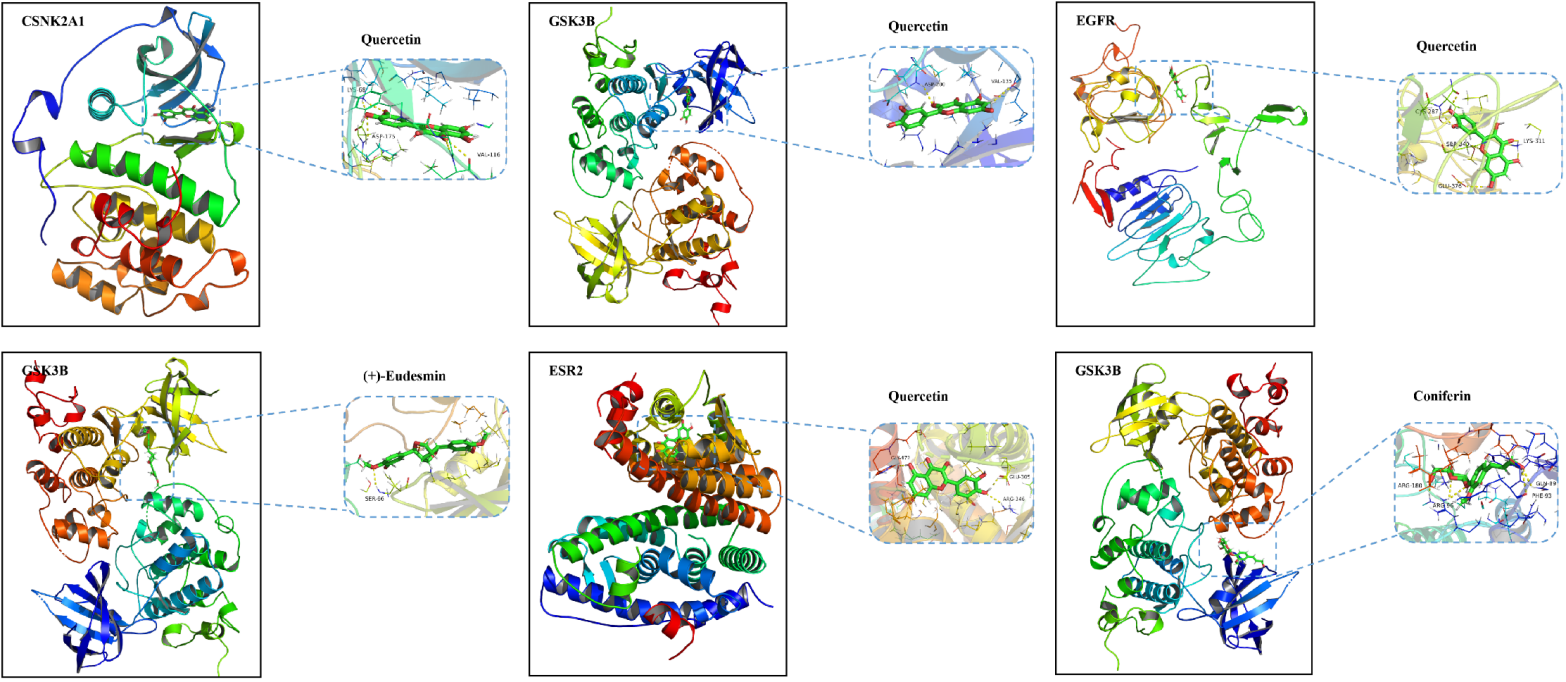
Molecular docking diagram of quercetin, (+)-eudesmin, and coniferin with related key targets.

To further explore the potential mechanism of AS for the treatment of AD, these nine key targets and their corresponding 16 compounds in AS were subjected to molecular docking operations. The results (**Table 3**) showed that the binding free energies of each compound docked to the proteins were all less than -5.0 kJ/mol, and the lower the free binding energy, the higher the affinity between the protein receptor and the small molecule ligand, and the more likely the interaction would occur. This indicates that the compounds in AS have high affinity with all relevant proteins. Docking results below -7.5 kJ/mol were visualized using Pymol software (**Figure 3**).

**Table 3:** Binding ability of key targets and corresponding compounds.

Target	PDB ID	Ligands	deltaG (kcal/mol)
CSNK2A1	1NA7	Quercetin	-8.7
GSK3B	1Q5K	Quercetin	-8.6
EGFR	1IVO	Quercetin	-8.2
GSK3B	1Q5K	(+)-Eudesmin	-7.7
ESR2	4J26	Quercetin	-7.7
GSK3B	1Q5K	Coniferin	-7.6
APP	1AAP	Quercetin	-7.5
GSK3B	1Q5K	Clausarin	-7.4
ESR1	1A52	(9Z,12E)-12-Nitrooctadeca-9,12-dienoic acid	-7.4
EGFR	1IVO	Coniferaldehyde glucoside	-7.4
CSNK2A1	1NA7	(9Z,12E)-12-nitrooctadeca-9,12-dienoic acid	-7.2
APP	1AAP	Caffeic acid	-6.8
ESR2	4J26	Caffeic acid	-6.4
GSK3B	1Q5K	Isofraxidin	-6.4
ESR1	1A52	Caffeic acid	-6.3
EGFR	1IVO	Isofraxidin	-6.3
ESR2	4J26	4-Hydroxycinnamic acid	-6.3
PAK1	3FXZ	Ciwujiatone	-6.2
ESR2	4J26	Ferulic acid	-6.2
ESR1	1A52	4-Hydroxy-3-methoxycinnamaldehyde	-6.1
ESR1	1A52	4-Hydroxycinnamic acid	-6.1
CFTR	1XMI	Ciwujiatone	-6.1
EGFR	1IVO	Caffeic acid	-6.1
ESR2	4J26	4-Hydroxy-3-methoxycinnamaldehyde	-6.1
ESR2	4J26	Ethyl gallate	-6.1
ESR2	4J26	Salicylic acid	-6.1
ESR2	4J26	3,4-Dihydroxybenzoic acid	-6.0
GSK3B	1Q5K	4-Hydroxy-3-methoxycinnamaldehyde	-6.0
APP	1AAP	Ferulic acid	-5.9
NTRK1	1HE7	Ciwujiatone	-5.9
EGFR	1IVO	(9Z,12E)-12-Nitrooctadeca-9,12-dienoic acid	-5.9
EGFR	1IVO	4-Hydroxy-3-methoxycinnamaldehyde	-5.8
EGFR	1IVO	Ferulic acid	-5.7
APP	1AAP	4-Hydroxy-3-methoxycinnamaldehyde	-5.4
EGFR	1IVO	Sesamol	-5.4

### 3.5 mRNA expression of key genes in mouse cerebral cortex

**Figure 4:**
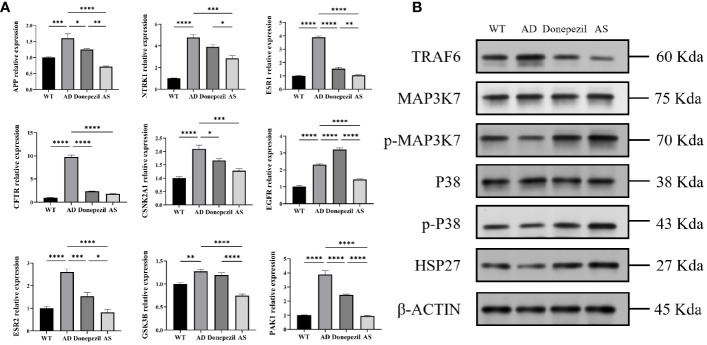
Results of qRT-PCR and Western blot (WB) about proteins related to the treatment of Alzheimer’s disease by *Acanthopanax senticosus*. **(A)** mRNA level of key targets on each group. **(B)** Results of WB (*n* = 3). ^*^ *P* < 0.05, ^**^ *P* < 0.01, ^***^P< 0.001, ^****^ *P*< 0.0001.

According to the statistical results of qRT-PCR (**Figure 4A**), the mRNA content expression of APP, NTRK1, ESR1, CFTR, CSNK2A1, EGFR, ESR2, GSK3B, and PAK1 in the hippocampus of the brains of mice in the AD group was significantly elevated compared with those of mice in the WT group (*P*< 0.01, *P*< 0.001, *P*< 0.0001). The mRNA expression of APP, NTRK1, ESR1, CFTR, CSNK2A1, EGFR, ESR2, GSK3B, and PAK1 in the AS group showed a significant decrease compared with that of the AD group (*P*< 0.001, *P*< 0.0001). It was demonstrated that the key targets of AS for the treatment of AD derived from network pharmacology were plausible and effective.

### 3.6 Western blot analysis of key pathways in the treatment of AD by AS

According to the results of the KEGG biological function analysis, the most relevant pathway for AS treatment of AD is involved in the MAPK pathway. The TRAF6, MAP3K7, p-MAP3K7, P38, p-P38, and HSP27 proteins in this pathway were selected for western blot analysis. The analysis showed that the expression level of TRAF6 protein in the hippocampal tissue of the AD group was significantly increased compared to that of the WT group, while the relative protein levels of p-MAP3K7/MAP3K7, the relative protein levels of p-P38/P38, and the expression of HSP27 protein were decreased. In contrast, in the hippocampus of mice after therapeutic intervention with AS, it can be found that mice in the AS group have a significant decrease in the expression level of TRAF6 protein and a notable increase in the expression level of MAPK phosphorylation and the HSP27 protein compared with the AD group; all the results were significant (**Figure 4B**). This suggests that AS may treat AD by regulating the phosphorylation process of the MAPK signaling pathway.

## 4 Discussion

It is established that the accumulation of extracellular amyloid β and neurofibrillary tangles in the brain contributes to the onset of Alzheimer’s disease; however, the current study suggests that AD is significantly linked to inflammatory processes within the central nervous system (CNS) (16). Flavonoid compounds have demonstrated the ability to inhibit neuroinflammatory processes and enhance memory recognition in mice with AD (17). According to the findings of clinical trials conducted by Tohda et al., the extract of AS is safe for augmentation in cognitive function, and it effectively alleviates anxiety in healthy individuals (18). In our study, we can see that AS has some efficacy in alleviating cognitive impairment caused by AD from the results of the behavioral experiments. In the open-field experiment, although there was no statistically significant difference between the groups of mice in terms of the distance traveled and the average speed of movement, the number of times the mice in the AS group crossed the central zone was significantly higher, which means that the mice showed good autonomous exploratory behavior and the negative emotional responses such as anxiety and nervousness were significantly alleviated by the administration of AS. According to the novelty recognition experiment, compared with the AD group, the percentage of time spent curiously exploring Z and the percentage of frequency of curious exploration increased in the AS group, while the percentage of number of times exploring Z was significantly higher in the donepezil group. Taken in combination, this suggests that both AS and donepezil can improve transient memory impairment in AD model mice.

To elucidate the potential therapeutic targets and mechanisms of action for AS in the treatment of AD, a comprehensive analysis of 245 candidate targets was conducted using network pharmacology techniques. A topological algorithm was employed to establish a protein–interaction network (PPI), which identified nine key targets for AS in AD treatment: amyloid-beta precursor protein (APP), neurotrophic receptor tyrosine kinase 1 (NTRK1), estrogen receptor alpha (ESR1), cystic fibrosis transmembrane conductance regulator (CFTR), casein kinase 2 alpha 1 (CSNK2A1), epidermal growth factor receptor (EGFR), estrogen receptor 2 (ESR2), glycogen synthase kinase 3 beta (GSK3B), and P21 activated kinase 1 (PAK1). The quantitative real-time PCR (qRT-PCR) data revealed that the mRNA expression levels of these targets in the hippocampus of AS-treated mice significantly decreased compared to that of the AD group. Several studies have shown that in addition to amyloid deposition and tau protein hyperphosphorylation leading to AD, mice carrying mutants of the human APP gene and lacking the apoE gene also exhibit memory deficits (19). It also promotes synapse formation, dendrite sprouting, and neuronal migration (20). Chronic intracerebroventricular injection of sAPPα in mice mitigated cognitive and synaptic deficits (21). NTRK1 (TrkA), a receptor for nerve growth factor (NGF), regulates neuronal growth, differentiation, and apoptosis in the CNS (22). Inflammatory mediators, including IL-1β, TNF-α, and IL-6, stimulate the synthesis of nerve growth factor (NGF) in both neurons and glial cells, concurrently increasing the expression of the TrkA receptor. Upon Toll-like receptor (TLR) activation, NGF binds to TrkA, triggering a cascade that involves the activation of Ras, PI3K, phospholipase Cγ1, and downstream signaling pathways, such as the MAPK pathway. This cascade interferes with intracellular TLR signaling, thereby augmenting the endogenous negative feedback mechanisms that modulate excessive inflammation (23–25). R Romano et al. showed that EGFR, part of the receptor tyrosine kinase superfamily, is crucial for neural stem cell maintenance, astrocyte maturation, and neurite outgrowth in the CNS (26). Its inhibition improves astrocyte proliferation after injury, enhances autophagy, and reduces Aβ toxicity and neuroinflammation (27), correlating with a reduced risk of associated dementia (ADD) (28). ESR1 and ESR2, estrogen receptor-related genes, are implicated in neuronal degeneration due to estrogen decline, leading to cognitive difficulties (29). ERα and ERβ expression in neurons and astrocytes is associated with cognitive function maintenance in older women (30, 31). CFTR, expressed in neurons and other cell types, is linked to improved cognitive performance with physical activity in CF patients (32, 33). GSK3, a ubiquitous serine–threonine kinase with two isoforms (GSK3α and GSK3β), is widely found in the CNS (34). GSK3β overexpression promotes the BACE1 cleavage of APP, favoring Aβ plaque formation, which disrupts the Wnt pathway, leading to tau phosphorylation and accelerating AD progression (34, 35).

According to the CTN graph, it is evident that the expression of key targets is associated with several anti-inflammatory and antioxidant properties in AS, including quercetin, (+)-eudesmin, 12-nitrooctadeca-9,12-dienoic acid, coniferaldehyde glucoside, caffeic acid, and isofraxidin, which has been shown to decrease Aβ production by inhibiting BACE1 and acetylcholinesterase (AChE), regulate the NF-κB pathway to reduce COX-2 levels, and mediate the inhibition of neuroinflammatory responses *via* the Nrf2/HO1 pathway, thereby intervening in AD (36–38).

The analysis of functional enrichment can facilitate a more comprehensive understanding of the interactions among gene products. According to the results of the KEGG analysis, the MAPK signaling pathway appears to be the most promising treatment avenue for Alzheimer’s disease (AD). Mitogen-activated protein kinases (MAPK) represent a group of serine–threonine kinases, comprising extracellular signal-regulated kinases (ERK), p38, and c-Jun NH2-terminal kinases (JNK) (39). Each MAPK signaling axis contains at least three components: MAPK kinase kinase kinase (MAP3K), MAPK kinase kinase (MAP2K), and MAPK kinase (40). Notably, the MAPK signaling pathway, which has been found to be significantly associated with AD development, can be triggered by inflammatory factors such as TNFα or IL-1β or in response to cellular stress (41, 42). Relevant studies have demonstrated that inhibiting the MAPK signaling pathway can effectively mitigate the inflammatory response, thereby alleviating the symptoms of AD (43, 44). The results of the Western blot analysis demonstrate that, compared with the AD group, the AS group exhibited a significant decrease in TRAF6 protein expression and an increase in p-MAP3K7/MAP3K7 relative protein content, p-P38/P38 relative protein content, and HSP27 protein expression levels. These findings suggest that AS may control the inflammatory response and improve cognitive dysfunction by inhibiting the expression of the TRAF6 protein, increasing the phosphorylation of the MAPK pathway and inhibiting the activation of pro-inflammatory factors.

In conclusion, AS can enhance short-term learning memory and effectively alleviate anxiety in 5xFAD mice. The mechanism of action is related to the activation of phosphorylation of the MAPK pathway and inhibition of the production of inflammatory factors. AS contains active compounds including quercetin, caffeic acid, and isofraxidin, with its main targets being APP, NTRK1, EGFR, GSK3B, and other genes. This study analyzes the feasibility and mechanism of action of AS in the treatment of AD, providing a novel approach to finding effective solutions for AD treatment in clinical settings.

## Data availability statement

The original contributions presented in the study are included in the article/supplementary materials, further inquiries can be directed to the corresponding author/s.

## Ethics statement

The animal study was approved by Animal Management and Use Committee of Shanghai University of Traditional Chinese Medicine. The study was conducted in accordance with the local legislation and institutional requirements.

## Author contributions

ZZ: Data curation, Validation, Writing – original draft, Investigation, Visualization. YW: Data curation, Visualization, Writing – original draft, Writing – review & editing. DS: Conceptualization, Resources, Writing – original draft, Funding acquisition, Writing – review & editing. CJ: Validation, Writing – original draft. HC: Data curation, Writing – original draft. FJ: Software, Writing – original draft. XB: Investigation, Writing – original draft. YS: Writing – review & editing. XS: Funding acquisition, Supervision, Writing – review & editing.
